# Soil Marginal Effect and LSTM Model in Chinese Solar Greenhouse

**DOI:** 10.3390/s24144730

**Published:** 2024-07-21

**Authors:** Weiwei Cheng, Changchao Wang, Yu Wang, Lirong Hao, Zhonghua Liu, Qingliang Cui

**Affiliations:** 1 College of Urban and Rural Construction, Shanxi Agricultural University, No.1, Mingxian South Road, Taigu District, Jinzhong 030801, China; chengweiwei280@sxau.edu.cn; 2 College of Agricultural Engineering, Shanxi Agricultural University, No.1, Mingxian South Road, Taigu District, Jinzhong 030801, China; wangchangchao2024@163.com (C.W.); haolirong0629@163.com (L.H.);

**Keywords:** solar greenhouse, soil thermal environment, high and low temperature boundaries, experimental study, numerical simulation

## Abstract

The food crisis has increased demand for agricultural resources due to various factors such as extreme weather, energy crises, and conflicts. A solar greenhouse enables counter-seasonal winter cultivation due to its thermal insulation, thus alleviating the food crisis. The root temperature is of critical importance, although the mechanism of soil thermal environment change remains uncertain. This paper presents a comprehensive study of the soil thermal environment of a solar greenhouse in Jinzhong City, Shanxi Province, employing a variety of analytical techniques, including theoretical, experimental, and numerical simulation, and deep learning modelling. The results of this study demonstrate the following: During the overwintering period, the thermal environment of the solar greenhouse floor was divided into a low-temperature zone, a constant-temperature zone, and a high-temperature zone; the distance between the low-temperature boundary and the southern foot was 2.6 m. The lowest temperature in the low-temperature zone was 11.06 °C and the highest was 19.05 °C. The floor in the low-temperature zone had to be heated; the lowest value of the constant-temperature zone was 18.29 °C, without heating. The minimum distance between the area of high temperature and the southern foot of the solar greenhouse was 8 m and the lowest temperature reading was 19.29 °C. The indoor soil temperature tended to stabilise at a depth of 45 cm, and the lowest temperature reading at a horizontal distance of 1400 mm from the south foot was 19.5 °C. The Fluent and LSTM models fitted well and the models can be used to help control soil temperature during overwintering in extreme climates. The research can provide theoretical and data support for the crop areas and the heating of pipelines in the solar greenhouse.

## 1. Introduction

According to the Global Food Crisis Report 2023, there is a severe global food crisis, with nearly 600 million people worldwide still at risk of food insecurity, vulnerability, and malnutrition by 2030 [1]. The food crisis has dramatically increased the pressure on agricultural resources. Solar greenhouses are effective in promoting sustainable agriculture, meeting the demand for food growth, and ensuring the supply of fruits and vegetables in areas with harsh winters [2]. Given its low energy consumption and high yield, the solar greenhouse is widely used by farmers in northern China [3]. In solar greenhouses, an inappropriate microclimate can lead to plant diseases and even death [4]. It is essential to know the greenhouse microclimate and its characteristics to maintain good conditions for crops [5]. There are environmental factors that affect the microclimate of solar greenhouses, including temperature and humidity [6,7,8], CO_2_ [9,10], light [11,12], and so on. Among them, soil temperature is an important environmental factor to ensure the growth of plant roots. Higher and low soil temperatures affect the growth of plant roots [13]; however, there are fewer studies on soil temperature [14]. Soil temperature is temporally delayed and complex [15]. Even a difference of 1 °C can have a significant impact on crop growth and development [16]. Therefore, to ensure a good growing environment for the crop, it is necessary to analyse the soil temperature.

In recent years, some scholars have studied the soil temperature field in solar greenhouses, and relevant studies have shown that soil temperature changes were mainly influenced by indoor air temperature, soil water content, and radiant energy received at the soil surface [17]. Fu et al. [18] used grey correlation analysis to compare soil temperature, air temperature, and total radiation at different depths to determine the correlation and noted that this correlation decreases with increasing soil depth [18]. The arch characteristics of the solar greenhouse have a significant effect on air temperature and solar radiation intensity, which indirectly affects soil temperature [19]. There was a significant vertical relationship between soil temperature and air temperature [20]. Soil water content influenced soil temperature variation [21,22]. Soil temperature was higher at sites with lower soil water content; shallow soil temperature especially was negatively correlated with soil water content [23].

The south-facing margin effect in solar greenhouses resulted in lower soil temperatures, which had a direct impact on crop growth on the south side [24]. The development and application of models is also essential for the understanding of internal environmental control in greenhouses. Regarding soil heat storage and exothermic models, Barbara [25] established a mathematical model of surface heat balance, which revealed a negative correlation between soil heat flux and indoor temperature by testing and analysing soil heat flux and soil temperature conditions in solar greenhouses in different regions. Canadian scholars have proposed a simplified model for forecasting the geologic initial temperature that further simplifies the process by analysing the correlation between geologic initial temperatures, annual air temperatures, and meteorological data [26]. A team of scholars has successfully predicted the annual soil temperature distribution across a simulated area by employing a heat transfer model with a finite element numerical solution methodology via FlexPDE software [27]. Related studies have also introduced numerical prediction models that consider the influence of variables such as shortwave solar radiation, thereby enhancing the accuracy of the predictions [28]. Furthermore, time-series-based modelling, in conjunction with feed-forward back-propagation neural networks and gene expression programming, presents a new avenue for the computation of soil temperature [29].

In conclusion, existing research on the greenhouse soil thermal environment encompasses a multitude of aspects, such as the influencing factors, the prediction modelling, and the research on the heat storage and release characteristics. Despite this, the mechanism of the stratified zoning of the soil thermal environment remains uncertain [30], and the stratified zoning boundaries are of significant importance with regard to the greenhouse heating range and to the crop cultivation area. In this study, temperature and humidity measurement points were arranged inside and outside the greenhouse and combined with CFD numerical simulation and a Python-based clustering algorithm to digitise and visualise data in order to explore the mechanism of changes in the soil thermal environment of the solar greenhouse and, thus, to define the optimal arrangement of measurement points for the purposes of soil temperature field partitioning and stratification.

## 2. Materials and Methods

### 2.1. Experimental Greenhouse

**Figure 1:**
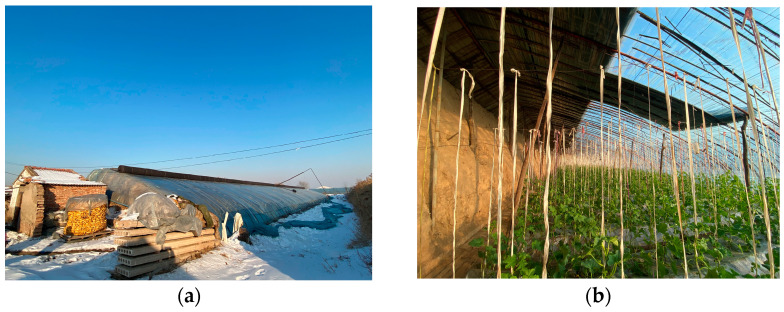
Inside and outside view of the experimental solar greenhouse. (**a**) Outside view of the experimental solar greenhouse; (**b**) inside view of the experimental solar greenhouse.

The experimental greenhouse was situated in the Taigu area of Jinzhong City, Shanxi Province (longitude 112°34′ E, latitude 37°25′ N) and had an average elevation of 870 m to 950 m. The climate is characterised by a hot, rainy summer and a cold, dry winter. The solar greenhouse as a whole is oriented 1.82 degrees to the southwest, with a length of 75 m, a span of 10 m, a ridge height of 4.2 m, and a back slope width of 1.4 m. The north wall is 3.3 m high and trapezoidal in shape, with a narrow top and a wide bottom. It has a width of 2 m at the top end and a width of 8 m at the bottom. In the period spanning December 2023 and February 2024, 31 days were characterised by clear skies, 23 days saw cloud cover, 13 days were marked by hazy conditions, and 6 days were affected by snow. The lowest temperature observed reached −23 °C, with the highest reaching 20 °C. Additionally, the maximum light intensity recorded at noon was 83,865 lx. The inner and outer structure of the solar greenhouse can be seen in Figure 1.

**Figure 2:**
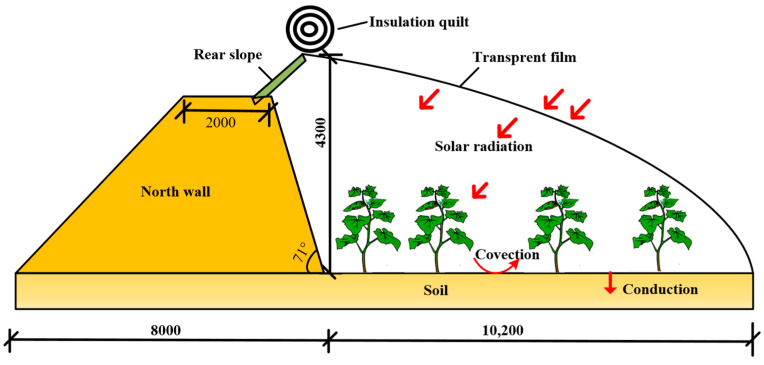
Radiation and dimensional diagram of the solar greenhouse.

The structure of this greenhouse is relatively simple, comprising mainly common building materials such as steel pipes, bamboo sheets, and wooden stakes. The east, west, and north walls are constructed of earth, with a 0.6-m pedestrian walkway situated within the greenhouse on the north side. The shed film is Crystal PO film, the top vent is near the top of the shed, and the bottom vent is at the bottom corner of the south side; only the top vent is opened in autumn and winter to achieve cooling and dehumidifying effect. From 8 a.m. to 10 a.m., insulation blanket was removed and solar greenhouse maintenance structures and crops could absorb solar rays; blankets were closed from 4 p.m. to 7 p.m., and heat was released from indoor floors and walls. The heat transfer diagram of a solar greenhouse is shown in Figure 2.

### 2.2. Mathematical Model

According to Liu and Jordan [31], for solar radiation illuminance on clear, cloudless days

(1) $$\beta_{s}=0.2710-0.2939\times \beta_{z}$$

(2) $$S=S_{0}\left(\beta_{z}+\beta_{s}\right)$$

From (1) and (2):

(3) $$S=S_{0}\left(0.7061\beta_{z}+0.2710\right)$$

According to Ma Chengwei et al. [32], it can be known that

(4) $$\eta_{s}=\eta_{0}(1-\theta /1000)(1-\gamma_{1})(1-\gamma_{2})(1-\gamma_{3})$$

(5) $$\eta_{z}=\eta_{0}\left[1-0.93^{\left(90-\theta \right)}\right](1-\theta /1000)(1-\gamma_{1})(1-\gamma_{2})(1-\gamma_{3})$$

Consider the effect of the opacity of the covering material in reducing the intensity of the direct light radiation and consider the conversion of some of the direct light into scattered light by the covering material.

(6) $$\begin{array}{ll} S_{Z} & =\beta_{z}S_{0}\times \eta_{z}\times (1-H_{aze})\\ & =\eta_{0}S_{0}\beta_{z}(1-H_{aze})(1-\theta /1000)\left[1-0.93^{\left(90-\theta \right)}\right](1-\gamma_{1})(1-\gamma_{2})(1-\gamma_{3}) \end{array}$$

(7) $$\begin{array}{ll} S_{s} & =\beta_{z}S_{0}\times \eta_{z}\times H_{aze}+\beta_{s}S_{0}\times \eta_{s}\\ & =\eta_{0}\beta_{z}S_{0}\left[1-0.93^{\left(90-\theta \right)}\right]H_{aze}(1-\theta /1000)(1-\gamma_{1})(1-\gamma_{2})(1-\gamma_{3})+\eta_{0}\beta_{s}S_{0}(1-\gamma_{1})(1-\gamma_{2})(1-\gamma_{3})\\ & =\eta_{0}S_{0}(1-\gamma_{1})(1-\gamma_{2})(1-\gamma_{3})[\beta_{z}(1-0.93^{\left(90-\theta \right)})H_{aze}+\beta_{s}]\\ & =\eta_{0}S_{0}\beta_{z}[H_{aze}-0.93^{\left(90-\theta \right)}H_{aze}-0.2939+0.2710/\beta_{z}](1-\gamma_{1})(1-\gamma_{2})(1-\gamma_{3}) \end{array}$$

And because

(8) $$\cos\theta =\cos\varphi \sinh_{s}+\sin\varphi \cosh_{s}cos(a-\gamma )$$

by (8).

(9) $$\frac{\cos\theta }{\cos\varphi }=\sinh_{s}+\tan\varphi \cosh_{s}cos(a-\gamma )$$

At the same time period, $h_{s},a,\gamma$ is a fixed value; as y increases, i.e., a point of the soil is getting closer to the north wall, the value of φ gradually decreases too. And 0°≤θ≤90°, according to Equation (9), can be seen to gradually decrease.

The following assumptions are made for scattered light:

According to the space-gray assumption, assuming that the scattered light is distributed throughout the solarium, the receiver of scattered solar radiation at a given point is the ratio of the volume of the polyhedron formed by a given point with the trellis film to the volume of the solarium.

Volume of the polyhedron formed by the point with the shed film = volume of the solarium − (volume of the polyhedron formed by the point with the east wall + volume of the polyhedron formed by the point with the west wall + volume of the polyhedron formed by the point with the north wall + volume of the polyhedron formed by the point with the contraction position of the quilt).

(10) $$\begin{array}{l} \omega =\frac{V-(S_{e}\times z-S_{w}\times (L-z)-S_{n}\times (W-x)-S_{m}\times h)/3}{V}\\ S_{e}=S_{w}=\frac{l_{1}+l_{2}}{2}\times h\\ S_{n}=L\times h\\ S_{m}=L\times w_{d}\\ \omega =\frac{6V-(l_{1}+l_{2}+2W+2w_{d}-2x)Lh}{6V} \end{array}$$

From (10), we can see that as it increases, it gradually increases.

Therefore, the intensity of solar radiation received at a point is as follows:

$$\begin{array}{ll} S & =\eta_{0}S_{0}\beta_{z}(1-H_{aze})(1-\theta /1000)\left[1-0.93^{\left(90-\theta \right)}\right](1-\gamma_{1})(1-\gamma_{2})(1-\gamma_{3})\\ & +\omega \eta_{0}S_{0}\beta_{z}[H_{aze}-0.93^{\left(90-\theta \right)}H_{aze}-0.2939+0.2710/\beta_{z}](1-\gamma_{1})(1-\gamma_{2})(1-\gamma_{3})\\ & =\eta_{0}S_{0}\beta_{z}(1-\gamma_{1})(1-\gamma_{2})(1-\gamma_{3})\left[\begin{array}{l} \left(1-H_{aze})(1-\theta /1000)(1-0.93^{\left(90-\theta \right)}\right)\\ +\omega (H_{aze}-0.93^{\left(90-\theta \right)}H_{aze}-0.2939+0.2710/\beta_{z}) \end{array}\right] \end{array}$$

As y increases, i.e., a point of the soil gets closer to the north wall, the θ-angle decreases gradually, ω increases gradually, all other parameters are fixed values, and the light radiation received by a point of the soil increases gradually, so that the closer it is to the north wall, the higher the soil surface temperature.



**Symbol**

**Parse**

**Unit**


$S_{0}$

Solar irradiance at the outer surface of the atmosphere

${W/m}^{2}$



S

Solar irradiance of light passing through the atmosphere to the outer surface of the greenhouse film

${W/m}^{2}$



$S_{z}$

Intensity of direct solar radiation received at a point indoors

${W/m}^{2}$



$S_{s}$

Radiant illuminance of scattered sunlight received at a point in the room

${W/m}^{2}$



$\beta_{Z}$

Atmosphere direct light transmittance %

$\beta_{S}$

Atmospheric transmittance of scattered light%

$\gamma_{1}$

Greenhouse construction shading losses%

$\gamma_{2}$

Loss of light transmittance due to ageing%

$\gamma_{3}$

Loss of light transmission due to ageing of the material%

θ

Angle of incident sunlight°

φ

Roof angle at any point°

$h_{s}$

Sun elevation angle°

α

Solar azimuth°

γ

Greenhouse azimuth°

$\eta_{0}$

Transmittance of direct light through dry clean new cover material at 0° incidence%

$\eta_{s}$

Scattered light transmission through cover materials%

$\eta_{z}$

Transmission of direct light through cover materials%

$H_{aze}$

Opacity of the cover material%

$S_{e}$

Eastern wall area

$m^{2}$



$S_{w}$

Western wall area

$m^{2}$



$S_{n}$

North wall area

$m^{2}$



V

Volume of the solar greenhouse

$m^{3}$



L

Greenhouse lengthm

W

Greenhouse spanm

h

Ridge heightm

$w_{d}$

Distance from quilt to back wallm

$l_{1}$

Top length of east and west wallm

$l_{2}$

Bottom length of east and west wallm

ω

Ratio of the volume formed by the interior point and the boundary of the greenhouse film to the total volume of the greenhouse%


### 2.3. Experimental Equipment

The soil temperature and humidity measurement instrument was selected from the Varitronix GSP-6 model, which complies with the GSP standard [33]. The instrument can be programmed to set upper and lower temperature thresholds, and will generate an alarm when the thresholds are exceeded. The device is capable of continuous testing for a period of five months, with a data storage capacity of 16,000 entries, with data recorded at 15 min intervals. The device exhibits a measurable temperature range of −40 °C to 85 °C. Its temperature accuracy is ±0.5 °C within a temperature range of 20 °C to 40 °C. Outside of this range, its accuracy is ±1.0 °C. The high precision of the test equipment minimized the occurrence of mechanical errors in the test measurement data.

The light measurement instrument is GS1, an industrial-grade wireless intelligent sensing device from Dalian Cloud Power Technology Co., Ltd. (Dalian, China). It is waterproof and dustproof with an IP65 grade, which effectively protects against the infiltration of dust and water droplets, and ensures the instrument runs stably. The sensor light range of the instrument is 0.01 to 157,000 lx, with an accuracy of ±10%. The instrument is capable of storing up to 300,000 data items in a continuous manner. It is also equipped with the capacity to transfer data via wireless networks and USB wired connections.

The measurement of air temperature was conducted using the RC-5 U-disk recorder from Jingchuang, which is certified in accordance with GSP [33] standards and incorporates a built-in NTC thermistor sensor with a resolution of 0.1 °C. The temperature measurement range is −30 °C to 70 °C, with an accuracy of ±0.5 °C within this range. Outside this range, the accuracy is ±1 °C, with a maximum capacity of 32,000 groups of data.

### 2.4. Test Methods

**Figure 3:**
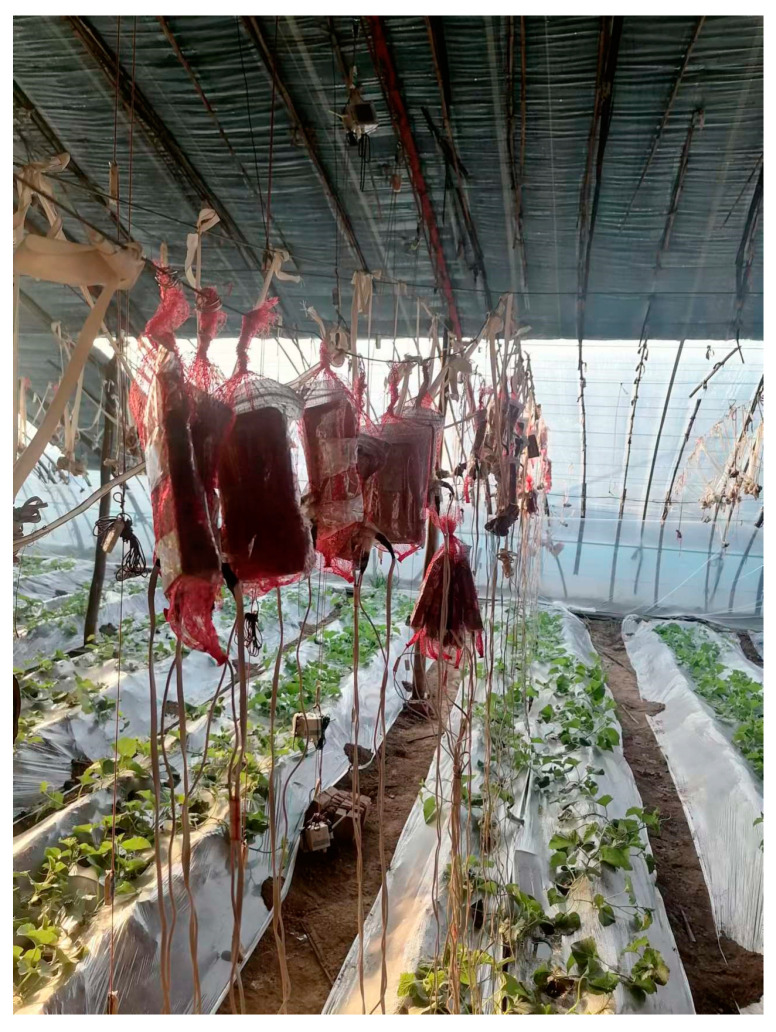
Schematic layout of measurement points.

The middle cross-section of the solar greenhouse was selected as the test cross-section, and the schematic layout of the measurement points is shown in Figure 3. Previous studies have indicated that the soil temperature in the area beyond 250 mm from the surface exhibits a gradual change [23]. This paper focuses on the variation of soil temperature in the field at a depth of 150 mm from the surface.

The middle section, extending from the east wall to the west wall of the solar greenhouse, was selected as the cross-section for the measurement point configuration. In this cross-section, the point of intersection between the arch and the soil was designated as point 0. The x-positive direction was defined as the direction from the upper surface of the soil to the downward direction, while the *y*-axis positive direction was defined as the direction pointing to the north wall, and the *z*-axis was defined as the positive direction from the east side to the west side of the greenhouse.

**Figure 4:**
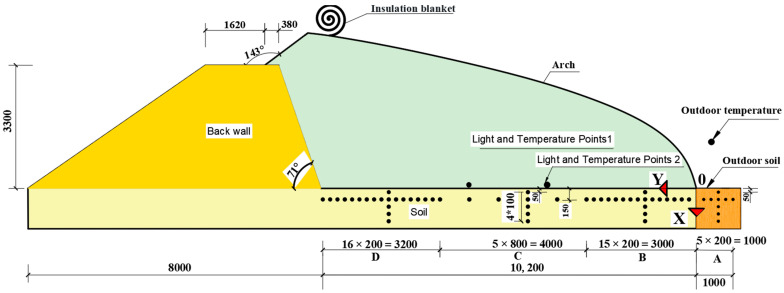
Layout of measurement points and cross-section of greenhouse structure.

The measuring points were arranged at z = 0 mm, i.e., the middle section of the solar greenhouse along the east–west direction was selected to arrange the measuring points. All measurement points were set at x = 150 mm. In the y direction, the regions were divided into four: A, B, C, and D. Region A was situated in the exterior of the greenhouse, with y located in the range of (−200, −1000) mm. There were five measurement points along the y direction, with a spacing of 200 mm between each point. Area B was situated within the central portion of the greenhouse, with a range of 200–3000 mm and a measurement interval of 200 mm. Area C was positioned inside the greenhouse and encompassed a range of 3000–7000 mm, with an inter-measurement distance of 800 mm. Area D was located within the greenhouse and fell within the range of 7000–10,200 mm, with a spacing distance of 200 mm between measurements. As shown in Figure 4.

The vertical measurement points in the areas A, B, C, and D were arranged in accordance with the following specifications: all measurement points z = 0 mm. The selected A, B, C, and D position y values were as follows: y = −600 mm, y = 1400 mm, y = 4600 mm, and y = 8400 mm. The four aforementioned positions were characterised by the arrangement of five measurement points along the x direction at 100 mm intervals. The x coordinates of these points were as follows: x = 50 mm, x = 150 mm, x = 250 mm, x = 350 mm, x = 450 mm.

Areas A, B, C, and D were situated in close proximity to the root system of the crop. In order to ascertain the light intensity at the soil surface within the greenhouse, two light measurement points were positioned in the vicinity of the crop, thus ensuring that the light received at ground level was not affected. Indoor lighting: two measurement points, X = 0, Y = 4000 mm, Z = −500 mm and X = 0, Y = 6200 mm Z = −500 mm. Outdoor lighting: without shade, height of 1.5 m at the arrangement of a light measurement point and air temperature measurement point.

### 2.5. Quartile Temperature Analysis

Quartiles are statistical measures that describe the distribution of data. The IQR (interquartile range) is the difference between the middle two quartiles; namely, the first quartile (Q1) and the third quartile (Q3).

The concept of outlier detection is based on the assumption that any value that falls below the 1st quartile minus 1.5 interquartile range (IQR) or exceeds the 3rd quartile plus 1.5 IQR may be considered an outlier. This method is employed to identify and remove extreme values from the data, which helps to reduce errors and biases in data analysis and ensures the quality of the dataset and the reliability of the analysis results.

Data screening for specific time intervals is a method based on time series analysis that focuses on analysing data at specific times of the day (e.g., nighttime). This is because different times may reflect different data characteristics. By classifying the data into different intervals (e.g., low-, medium-, and high-temperature intervals) according to the magnitude of their values, the characteristics and differences of the data within each interval can be analysed in greater detail.

In this study, soil temperature was measured at 15 min intervals over a period of 15 weeks, from 25 September 2023 to 15 April 2024. The data from the nighttime period between 1 December 2023 and 3 March 2024 were also included. A dataset was constructed using Python, which was employed to develop an outlier processing methodology based on interquartile spacing (IQR) and a classification method based on the quartiles of the temperature data. This was used to identify the boundary points of overwintering soil partitioning and the minimum and maximum values of different months in Jinzhong, Shanxi Province.

### 2.6. CFD Numerical Simulation

The CFD method is frequently employed to model the temperature field in solar greenhouses. This paper utilised experimental data as boundary conditions to conduct a CFD simulation study, with the objective of elucidating the partition stratification phenomenon and the marginal effect phenomenon of soil thermal environments in solar greenhouse.

#### 2.6.1. Grid Division

**Figure 5:**
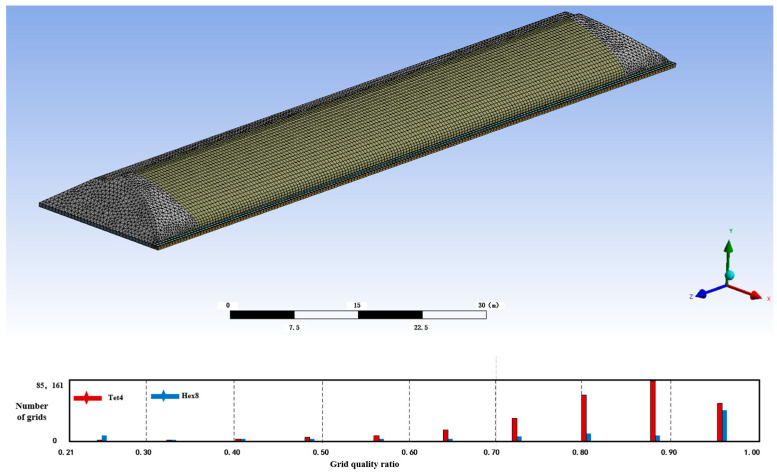
Grid quality.

A three-dimensional model of the solar greenhouse was established in SOLIDWORKS 2016, and the grid was divided using Fluent 19.0 for numerical calculations. The grid size of the three-dimensional model was set to 0.5 m, the internal size to 0.4 m, and the resulting three-dimensional model after the division of the grid is shown in Figure 5.

In accordance with the aforementioned criteria, the majority of grids were found to have a cell mass of 0.84 or greater. Furthermore, over 90% of the grids exhibited a cell mass of less than 0.5 m. The model in question was constructed entirely from 315,931 grid cells, with no “negative mesh” errors, and was in compliance with the requisite specifications of the FLUENT simulation testing.

#### 2.6.2. Boundary Condition

This study analysed the condition of the indoor air temperature field and soil temperature stratified zoning, which has been set up as a transient model considering the energy equation and a DO solar radiation model. The temperature values of each boundary, including the backslope, soil, and fluid domains, were defined separately with the experimental data. The initial temperature values of each soil boundary are shown in Table 1.

For the other boundaries, the initial temperature value was 21 °C and the temperature was locally initialised for the areas requiring accurate solutions to provide the best fit to the actual conditions of initial temperature change. Appropriate material parameters were selected and the thermophysical properties of each material are shown in Table 2. According to the thermophysical properties of the materials and the boundary conditions, 18:00 of a day to 8:00 of +1 day were selected to numerically calculate the entire computational domain to obtain the temperature changes in each region for 14 h.

### 2.7. LSTM Model

Long Short-Term Memory (LSTM) networks can be utilised to address the problem of vanishing or exploding gradients, which enables them to effectively model long-term patterns in practice.

The introduction of a distinctive gating mechanism has enabled LSTM to overcome the limitations of traditional modelling approaches, significantly enhancing the learning and representation of time-series data. This has made LSTM an indispensable and pivotal component in the field of modern deep learning.

The purpose of this study was to investigate the effect of soil on temperature within solar greenhouses. This was done by measuring soil temperature at various times and by utilising the Long Short-Term Memory (LSTM) model to learn and predict soil temperature. The findings of this study can be used as a foundation for future research into the control of soil temperature in greenhouses.

The fundamental principle underlying the design of the Long Short-Term Memory (LSTM) cell state lay in its capacity to regulate the flow of information in a dynamic and adaptable manner, throughout the sequence in question. The cell state traversed the entire sequence, enabling the storage and erasure of information over an extended period. LSTM comprised three essential gating units. The Input Gate, Forget Gate, and Output Gate collectively determined the update of the cell state as well as the final output of the hidden state.

The gating units generated values between 0 and 1 through the application of a sigmoid function, which represented the degree of acceptance of new information, the degree of forgetting of old information, and the degree of exposure of the cellular state to the output, respectively.

Oblivion Gate: The information in the cellular state of the previous moment that was to be forgotten was identified. The forgetting gate activation value, *f_t_*, was computed from the current input, $x_{t}$, and the hidden state of the previous moment, $h_{t-1}$, through a fully connected layer with a sigmoid activation function.

$$f_{t}=\sigma \left(W_{f}\cdot \left[x_{t},h_{t-1}\right]+b_{f}\right)$$

The input gate determined which information in the input at the current moment should be added to the cell state. The input gate comprised two components. The first was to determine the admission weight of the information, which was achieved through the sigmoid function. The second was to compute the candidate state, $C_{t}$, through the tanh function.

$$\begin{array}{c} i_{t}=\sigma \left(W_{i}\cdot \left[x_{t},h_{t-1}\right]+b_{i}\right)\\ {\tilde{C}}_{t}=\tanh\left(W_{C}\cdot \left[x_{t},h_{t-1}\right]+b_{C}\right) \end{array}$$

A cell state update was initiated by combining the results of the forgetting gate and input gate to update the cell state $C_{t}$.

$$C_{t}=f_{t}⊙C_{t-1}+i_{t}⊙{\tilde{C}}_{t}$$

The output gate determined which information in the cell state should be passed on to the hidden state at the next moment or as model output at the current moment. The activation value $O_{t}$ of the output gate is computed from the current input $x_{t}$ and the hidden state $h_{t-1}$ at the previous moment through a fully connected layer with a sigmoid activation function, and then multiplied element-wise with the value of the cellular state after the tan*h* function to obtain the final hidden state $h_{t}$:

$$\begin{array}{l} O_{t}=\sigma \left(W_{0}\cdot \left[x_{t},h_{t-1}\right]+b_{0}\right)\\ h_{t}=o_{t}⊙\tanh(C_{t}) \end{array}$$

## 3. Results and Discussion

In this paper, through experimental research, the Python-based cluster analysis method, and the CFD numerical method, the article analysed the law of change of soil temperature in different spans and different depths of daytime greenhouse, determined the boundary point of soil temperature field zoning and layering, analysed the mechanism of change of soil thermal environment, and provided theoretical support for improving the soil spatial utilization rate and crop growth.

### 3.1. Indoor and Outdoor Light and Thermal Environment

#### 3.1.1. Changing the Indoor and Outdoor Light Intensity Rule in Different Months

**Figure 6:**
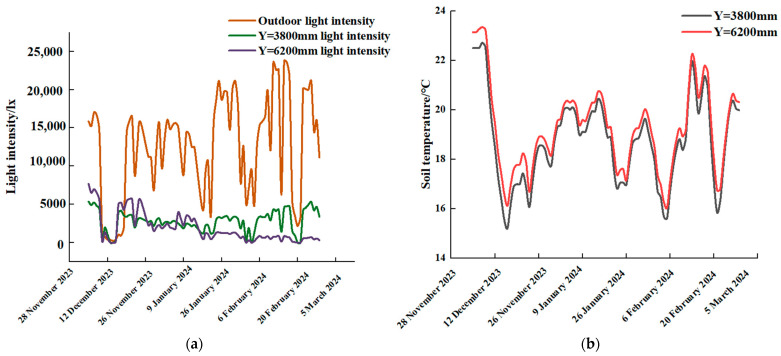
Variation of light intensity and soil temperature. (**a**) Light intensity inside and outside the greenhouse; (**b**) soil temperature.

We selected an outdoor measuring point, two indoor light measuring points (X = 0 mm and Z = −500 mm), two measuring point y values of 3800 mm and 6200 mm, and light intensity data for processing on 5 December 2023–15 April 2024; at the same time, we selected two indoor measuring points (X = 150 mm and Z = 0 mm) and two measuring point y values of 3800 mm and 6200 mm on the temperature of the ground measuring point for processing. The rule of change of law is shown in Figure 6.

As can be seen from Figure 6, most of the period of outdoor light intensity was significantly higher than the indoor, and the shed film on the light radiation blocking effect is obvious. A small part of the period of outdoor light intensity and indoor light intensity was approximately equal; this is due to the period of snow-covered outdoor measuring points and the roller shutter being in the state of covering the shed film. y = 3800 mm and y = 6200 mm on the light intensity throughout the observation period of the light intensity is approximately equal to the change trend, which was the same. y = 6200 mm on the soil temperature value was higher than y = 3800 mm on the soil temperature value. The closer the distance from the north wall, the better the insulation effect, which is the same as the previous study [34]. There was a seasonal decrease in light intensity during the winter months, which lasted until mid-January. This was related to the geographical location of the area, the shorter hours of sunshine, and the lower intensity of solar radiation.

#### 3.1.2. Impact of Span Changes on Interior Lighting

There were typical sunny weather conditions for the selected day, 4 January 2024, with the blankets put away at 8.55 a.m., the vents opened at 10.45 a.m., the vents closed at 3.35 p.m., and the blankets lowered at 4.17 p.m.

**Figure 7:**
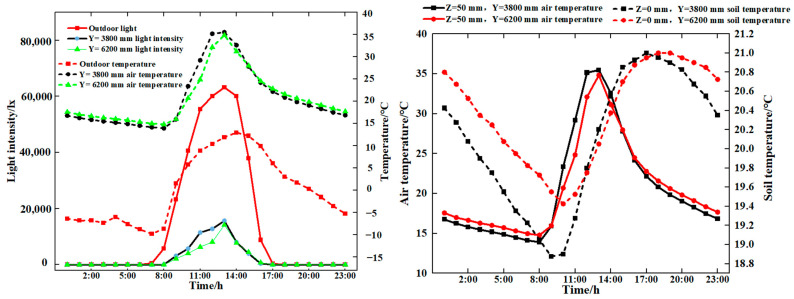
Variation of light intensity, air temperature, and soil temperature at different locations (4 January 2024).

We selected outdoor and indoor air temperature measurement points of Z = 0.5 m, Y = 3800 mm and Z = 0.5 m, Y = 6200 mm at the measurement point temperature analysis to explore the shed membrane on the outdoor light blocking efficiency, span distance on the indoor light intensity of the extent of the impact and the vent on the indoor heat storage, and its light intensity and temperature change rule as shown in Figure 7.

As can be seen from Figure 7, the indoor and outdoor measurement points of light intensity and air temperature change rule of law was the same, the highest value of temperature time lagged behind the maximum value of light, the indoor temperature was higher than the outdoor temperature, and the greenhouse insulation effect was better. In the morning hours, the soil temperature minimum lagged behind the air temperature minimum in time because, on the one hand, the soil temperature was still higher than the air temperature during the period when the blankets were put away and, on the other hand, the evaporation of soil moisture removed some of the heat.

During the period from 8.55 a.m. to 10.45 a.m., when the quilts were put away but the air vents were not opened and the air and floor were in a state of absorbing solar radiation, the difference between the integral of the light intensity at the indoor and outdoor measuring points and the corresponding temperature at the measuring points was calculated. The sum of outdoor light intensity was 504,770.5 lx, and the temperature increased by 9.5 °C; the sum of light intensity at Y = 3800 mm was 68,514.2 lx, the light intensity was 0.135 times the outdoor light intensity, and the air temperature increased by 10.176 °C. The sum of light intensity at Y = 6200 mm was 46,645.50 lx, the light intensity was 0.092 times, and the air temperature increased by 6.857 °C. The soil temperature did not change during this period, mainly due to crop leaves shading the soil measurement point and its corresponding measurement point receiving less direct radiation. The indoor light intensity was significantly lower than the outdoor light intensity; in the winter, farmers should be used to reflective film on the crop area of replenishing light operation. The closer to the north wall during this time period, the greater the light intensity and the greater the change in air temperature, but the smaller the change in soil temperature.

During the period from 10:45 to 15:35, when the air vents were open and the duvets were not lowered, the difference between the integral of the light intensity and the corresponding temperature at the indoor and outdoor measuring points was calculated. The total light intensity received at the outdoor measurement point during the specified time period was 279,442.4 lx, and the outdoor air temperature increased by 5.9 °C. The total light intensity received at Y = 3800 mm was 52,740. A light intensity at a distance of 49.6 lx and an air temperature increase of 2.5 °C was observed at this location. The light intensity at a distance of 6200 mm was 408,778.2 lx, with an air temperature increase of 5.4 °C. The soil temperature exhibited a more rapid increase during this stage, reaching a maximum of 1.9 °C. This was primarily due to the fact that the indoor soil was in a heat-absorbing state, while the amount of sun-scattered radiation was greater. Additionally, the air was in a heat-absorbing, convective, and conductive state, and light inhomogeneity had a more pronounced effect on air temperature. Furthermore, ventilation had a greater impact on the rise of indoor air temperature.

During the period between 15:35 and 16:17, the vents were closed and the quilts were not lowered. The total light intensity received outdoors during this period was 81,565.43 lx, and the outdoor air temperature decreased by 1.647 °C. The light intensity received at a distance of 3800 mm was 16,274.58 lx, and the air temperature decreased by 2.502 °C. The light intensity received at a distance of 6200 mm was 21,692.79 lx, and the air temperature decreased by 1.989 °C. The soil temperature remained relatively stable throughout the observation period, largely due to a reduction in light intensity and the subsequent low levels of scattered radiation reaching the soil.

A comparison of light intensity at different locations inside and outside the greenhouse revealed that in low light conditions, the indoor light intensity is approximately 0.09 times that of outdoor light. In strong light conditions, the span can be adjusted by adjusting the angle of incidence of the light to enhance the absorption of light intensity and improve the solar energy utilisation rate.

### 3.2. Soil Temperature

#### 3.2.1. Rule for Measuring Soil Temperature at a Point with the Same Burial Depth and Different Spans

**Figure 8:**
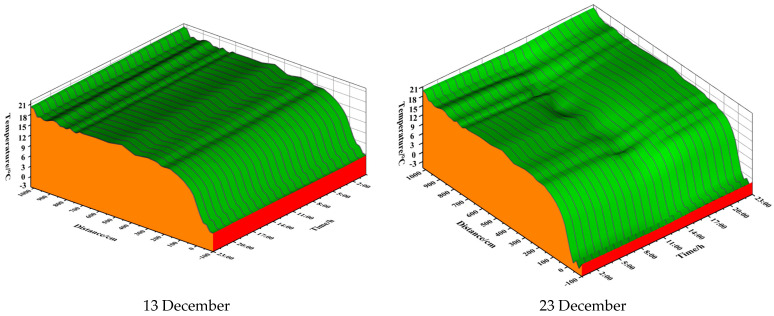
Soil temperature variations.

The weather conditions of 13 December 2023, with light snowfall, and 23 December 2023, with clear skies, were selected as typical for the investigation of the changing pattern of soil temperature with different spans of soil burial depth, as shown in Figure 8.

As can be seen from Figure 8, the same burial depth (*x* = 150 mm) and temperature range *y* = (−1000, 10,200) mm were maintained throughout the entire period. However, as the depth increased, the temperature gradually increased as well. On the same day, the maximum difference was less than 3 °C, and the magnitude of change in the measuring point temperature was less than the change in the span of the temperature. Therefore, it was necessary to analyse the temperature of the soil measuring point in accordance with the change rule of the span of the temperature.

#### 3.2.2. Determination of Soil Thermal Environment Zoning Boundary Points

**Figure 9:**
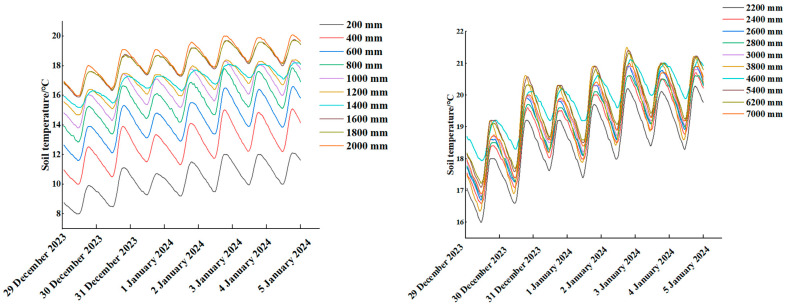

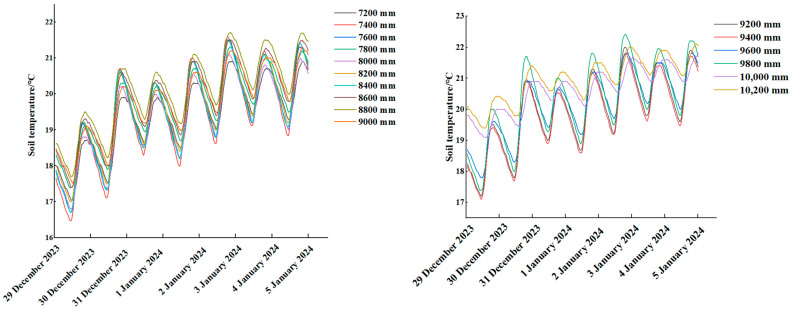
Plot of soil gauge temperatures over time for the period 29 December 2023–5 January 2024.

The data from 30 December 2023 to 5 January 2024 were selected. The vents were not opened between 30 December 2023 and 2 January 2024. The period between 10:44 p.m. and 11:40 p.m. was excluded from the analysis. From 3 January 2024 to 5 January 2024, the upper vent was opened, and the quilt was lowered at approximately 3:30 p.m. The variation of X = 15 cm soil temperature over time for seven consecutive days was analysed, as shown in Figure 9.

As can be seen from Figure 9, the soil temperature exhibited two distinct trends: a rise and a decline. The maximum and minimum values of soil temperature exhibited a time lag phenomenon, which persisted despite the opening of the vents. Therefore, the determination of the boundaries between high and low soil temperatures was appropriate for the application of the two-segment method, whereby two periods of time are considered, respectively, for the covering of the quilt without a light section and no quilt cover to receive radiation.

The temporal interval between 4 January 2024 and 5 January 2024, for instance, encompasses a period of darkness from 17:00 on 4 January 2024 to 8:00 on 5 January 2024. The downward trend in soil temperature and the exothermic state of the soil are indicative of the period with light, which occurs from 8:00 to 17:00 on 5 January 2024. This is when the greenhouse removes its quilt, allowing the soil to receive solar radiation.

**Figure 10:**
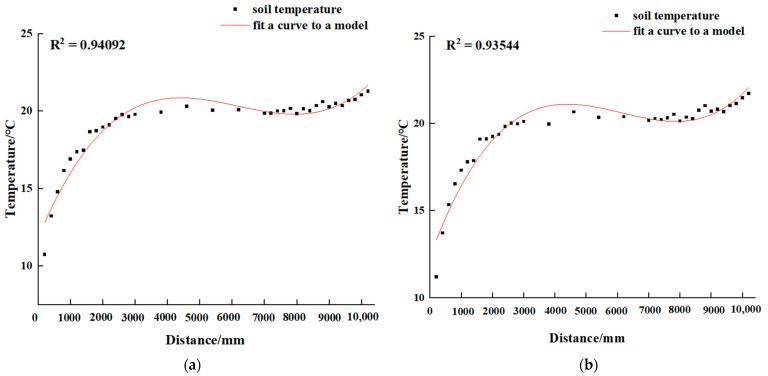
Scatter plots and fitted curves under different light conditions. (**a**) Periods with light; (**b**) no-light period.

The data from the two time periods were subjected to analysis and processing, with each measurement point in the two time periods averaged. Scatter plots were created, with the distance of each measurement point from the coordinate origin represented on the horizontal axis and the temperature on the vertical axis. A suitable type of trend line was added, according to the characteristics of the data. Fitting was done using a cubic function and fitted well. The coefficient of determination (R^2^) of the trend lines was greater than 0.93, indicating that the mathematical model simulation effect is satisfactory. This is illustrated in Figure 10.

As can be seen from Figure 10, a discernible three-segment trend in soil temperature within the greenhouse was evident, which aligned with the findings of Sun et al. (2012) on soil temperature. The fit function, which has been derived from the data, was also a three-order function. The function was divided into three distinct regions: a rising region, a levelling-off region, and a rising again region. These were defined as the low-soil-temperature region, the constant-soil-temperature region, and the high-soil-temperature region. The maximum discrepancy between the temperature of the measuring point in the gentle zone of this function in the state of covering the film and in the state of receiving radiation was 0.37 °C and 0.33 °C, which is less than the error of the equipment.

A method for determining the Lower Temperature Area Boundary Point (LTABP) and the Higher Temperature Area Boundary Point (HTABP) was the equation used to determine the maximum boundary point of the low-temperature zone and the minimum boundary point of the high-temperature zone, as well as to find the average value of the temperature of the measurement points in the region of these two boundary points, which is used as the temperature value of the constant temperature zone. A difference of only 1 °C in the soil temperature may have a significant effect on crop growth and development [16], taking account of the potential for error in the measuring equipment of up to ±0.5 °C. The mean value of the temperature in the constant-temperature zone, minus 0.5 °C, was taken as the initial point of the constant-temperature zone, which represented the upper limit of the low-temperature zone. Conversely, the mean value of the temperature in the constant-temperature zone, plus 0.5 °C, was considered the final point of the constant-temperature zone, which represented the lower limit of the high-temperature zone. The corresponding temperature value was incorporated into the proposed function in order to identify the low-temperature boundary point and the high-temperature boundary point.

This study presents the high- and low-temperature boundary points for the time period 4 January 2024 18:00–5 January 2024 18:00, for time periods with and without light.

Light time periods: A simulation of the phenomenon under consideration revealed that the temperature reached its highest value at a distance of y = 4600 mm, with a temperature value of 20.32 °C, and that a maximum temperature difference of 9.42 °C was observed over the range of Y = (0, 4600) mm. It showed a rising at y = 8000 mm, with a temperature value of 19.85 °C and a maximum temperature difference of 1.46 °C in the range of Y = (8000, 10,200) mm. The maximum temperature difference in the y = (4600, 8000) mm was 0.47 °C, which was less than the error of ±0.5 °C of the device. The mean temperature value within the specified range (4600 mm–8000 mm) during the light exposure period was determined to be 20.03 °C. The maximum temperature observed in the low-temperature region was 20.03 ± 0.5 °C, while the minimum temperature recorded in the high-temperature region was 9750 mm. These values were subsequently incorporated into the function to derive y = 2430 mm and y = 9750 mm. The low-temperature region with a light time period was defined by the range y = (0, 2430) mm, the high-temperature region was y = (9750, 10,200) mm, and the mean value of temperature in the constant-temperature region is 20.03 °C.

Concurrently, the low-temperature cut-off point for the no-light time period was at y = 2640 mm, while the high-temperature cut-off point was at y = 9930 mm. The mean temperature in the constant-temperature region was 20.61 °C.

#### 3.2.3. Distribution of Soil Temperature Boundary Points in Different Months

**Figure 11:**
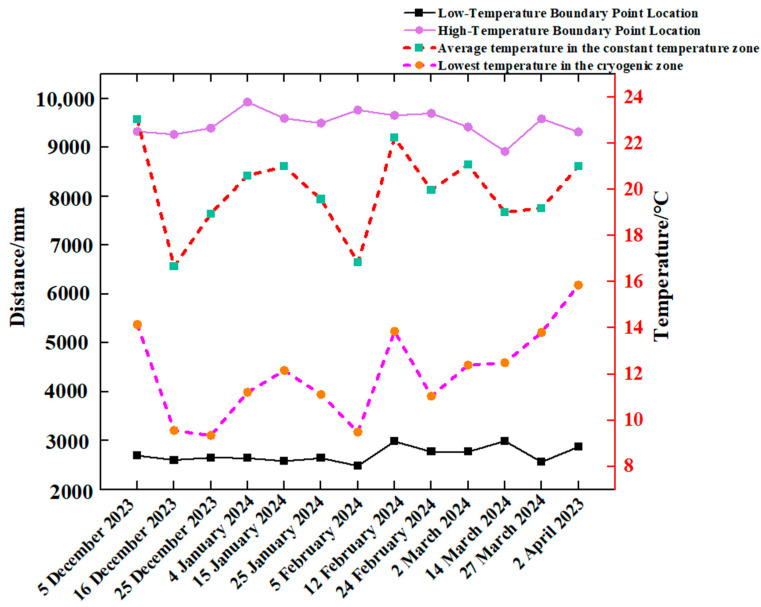
Seasonal variation of soil temperature thresholds at X = 150 mm depth in solar greenhouse.

We attempted to define the end point of the low-temperature zone and the beginning of the high-temperature zone for different months, according to the methodology for determining the high- and low-temperature boundary points of the soil in Section 2.5. In order to ascertain the impact of the overwintering period on the soil temperature, data were collected over a period of more than four consecutive months (December 2023–early April 2024). These data were then used to determine the daily periods of no light based on the values from the light instrument. The high- and low-temperature boundary points for each nighttime period in the absence of light must be identified, after which the two points can be divided into different temperature zones of the soil at night. The distribution of the high- and low-temperature boundary points for different months is presented in Figure 11.

As can be seen in Figure 11, the boundaries of the soil’s high- and low-temperature zones in the greenhouse exhibited notable variation throughout the period between December 2023 and April 2024. However, the magnitude of these changes was relatively modest; the low-temperature boundary point was located at (2480, 2990) mm, with a temperature minimum of 9.33 °C and a maximum of 15.85 °C, and the high-temperature boundary point was located at (8920, 9930) mm, with a temperature minimum of 17.16 °C. The minimum temperature in the constant-temperature zone was 16.66 °C.

The horizontal distance between the low-temperature boundary point of the soil in the greenhouse and the south/bottom corner was 2480 mm–2990 mm. This distance was compared with the marginal effect boundary point at 1600 mm for sunken solar greenhouses [35], which indicates that the marginal region is larger for non-sunken solar greenhouses.

Liu Zhengjia et al. [36] posited that the optimal soil temperature for crop growth in agricultural fields is 24.8 °C. Previous studies have indicated that the tomato root system is moderately inhibited when the soil temperature is 13 °C, and slightly inhibited when the soil temperature is 16 °C [37]. Additionally, Hongyun et al. [38] concluded that the root system of melon seedlings was restricted at 8 °C to 15 °C. Therefore, in the solar greenhouse in Jinzhong, Shanxi Province, the low-temperature zone of the soil should be heated under the condition of covering the film at night.

### 3.3. Soil Thermal Environment at Different Depths

**Figure 12:**
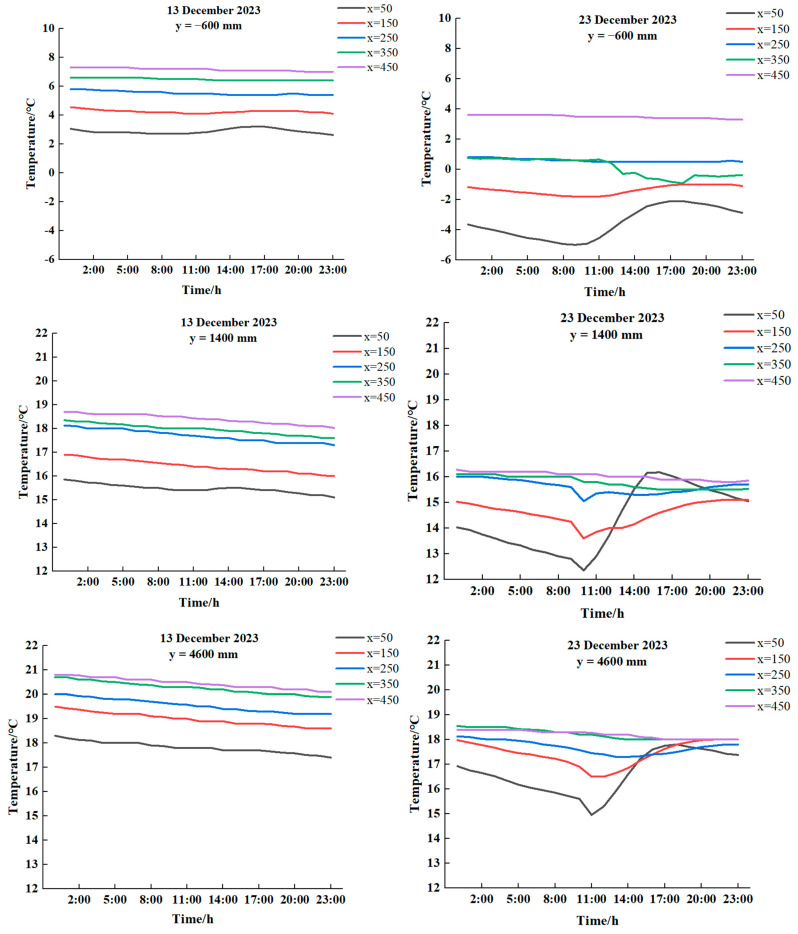

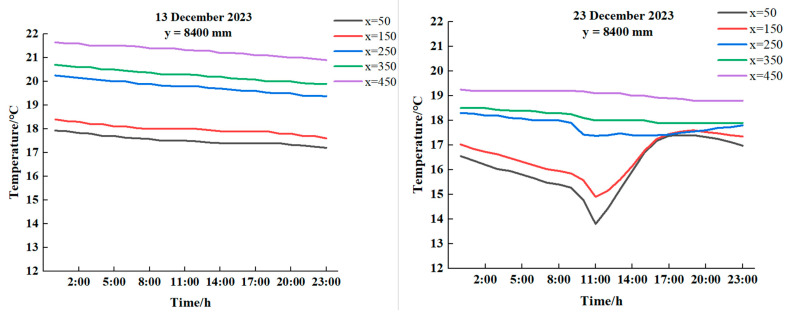
Variation in *x* direction for different span positions (13 December 2023 and 23 December 2023).

In order to elucidate the modification of soil temperature at varying depths in distinct span positions, the circumstances under sunny and weak light weather conditions were selected for analysis. The soil temperatures at varying depths in distinct span positions on the dates 12 December 2023 (weak light) and 23 December 2023 (sunny) were analysed, as illustrated in Figure 12.

From Figure 12, it can be seen that in spite of the weather conditions, the indoor and outdoor soil temperatures increased gradually with depth, with the greatest increase occurring at a depth of *x*. The outdoor temperature (y = −600 mm) exhibited a high degree of variability; a comparison of the temperature change of soil measurement points at x = 350 mm and x = 450 mm with the same span indicated a gradual decrease in the trend, with the exception of a maximum difference of 1 °C at y = 8400 mm. Furthermore, the temperature of these two points at other span positions in the room is less than 0.5 °C. With the exception of the maximum difference of 1 °C at y = 8400 mm, the temperature at these two points is less than 0.5 °C at all other span positions in the solar greenhouse. At the same depth, the soil temperature gradually increased as the y value increased, which indicated that the measuring point was situated in close proximity to the north wall. It has been observed that temperature changes in shallow soils exhibit significant fluctuations during periods of peak light intensity. In contrast, deeper soils, particularly those found in greenhouses, were not affected by light intensity and exhibit flatter temperature changes.

With regard to the magnitude of soil temperature change, soil temperature can be divided into three layers along the x direction: The upper layer of the soil, spanning a depth of 0–50 mm, was significantly affected by fluctuations in air temperature. In the second layer, which extended from 50 to 350 mm, a buffer layer was present, which was subject to convection currents in the air and also to heat transfer from the deeper soil, where temperature changes began to be more moderate. The third layer, with a depth of 350–450 mm, was characterised by its ability to stabilise the environment. This was achieved by maintaining a relatively stable temperature conduction and resisting large fluctuations in light intensity.

### 3.4. Quartile-Based Clustering Results

A thorough examination of the distribution of soil temperatures within the greenhouse was conducted by selecting and analysing data from December 2023 to March 2024, with a focus on the period between 18:00 and 8:00 the following day. A Python-based quartile approach was employed to classify the collected nocturnal soil temperature data. This study commenced with the standardisation of four consecutive months of soil temperature data from 41 monitoring devices. The data were then filtered between the hours of 18:00 p.m. and 8:00 a.m. the following day, with the average temperature at each measurement point calculated for the aforementioned time period. Subsequently, cluster analyses were conducted in order to categorise the measurement points into low-, constant-, and high-temperature zones. The clustering results were presented in tabular format, as shown in Table 3.

As can be seen from Table 3, during the overwintering period, the distance between the termination point of the low-temperature region of the solar greenhouse and the foot of the south side was 2600 mm. The lowest temperature in this zone was 11.06 °C, while the highest temperature was 19.05 °C. It is recommended that the soil in the low-temperature zone be heated in extreme weather. The minimum temperature in the constant-temperature zone was 18.29 °C, while the minimum temperature in the high-temperature zone was 19.29 °C. It was determined that there was no need to warm the constant- and high-temperature zones during the overwintering period.

### 3.5. CFD Numerical Analysis Results

**Figure 13:**
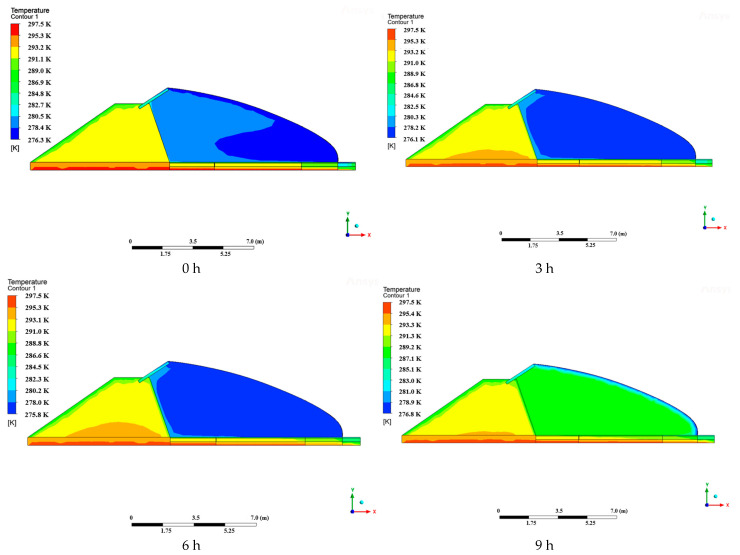
Cloud view of indoor temperature at different times.

In order to investigate the change rule of soil and air temperatures inside and outside the greenhouse over time, a geometrical model of the test greenhouse was created and simulated using Fluent19.0 software. This was done in order to investigate the partitioning and stratification of soil temperatures. The data were selected from a sunny day on 23 December. The vents were not opened, the quilts were covered between 0:00 and 9:00, solar radiation was received by the quilts between 9:00 and 16:00, and the quilts were put down at 16:00. The air and soil temperatures under the condition of covering the sheet during the 0:00–9:00 time period were simulated as a whole, and the resulting cloud plots of the intermediate cross-section in different time periods are shown in Figure 13.

From the simulation cloud diagram 13, it can be seen that with the increase of time, the soil, wall, and air temperatures show a decreasing trend, and these three parts of the temperature show a difference in space. The air temperature and wall temperature in the components of the boundary of the low-temperature trend, the soil temperature along the lateral and vertical subdivision block trend, the soil temperature outside the greenhouse, and the greenhouse soil temperature show a discontinuous upward trend; before and after the greenhouse, there is a clear division and stratification phenomenon. With the passage of time, the soil temperature stratification phenomenon gradually weakened.

In the case of covering the blanket, the temperature of the soil and the north wall was significantly higher than the air temperature. At this point, the soil and north wall were exothermic, releasing heat to maintain the greenhouse temperature. At this point, the soil temperatures exhibited significant zoning; stratification was observed, with the outdoor soil temperatures significantly lower than the indoor soil temperatures.

Due to the difference between the indoor soil temperatures and outdoor air temperatures, the indoor soil temperatures were higher near the north wall. This resulted in a more pronounced difference in soil temperature, resulting in more heat being released from the soil near the south side of the footing. There were several reasons for the higher soil temperatures on the north wall. Firstly, the height of the arch increases as the north wall rises, making it more difficult for the soil to release heat. Secondly, the north wall also has an exothermic wall effect, which further increases the soil temperature. As a result, both ground and air temperatures are higher near the north wall.

### 3.6. LSTM Model

The objective of this study was to develop and test LSTM models to predict the point data for overwintering in the period between December 2023 and April 2024.

**Figure 14:**
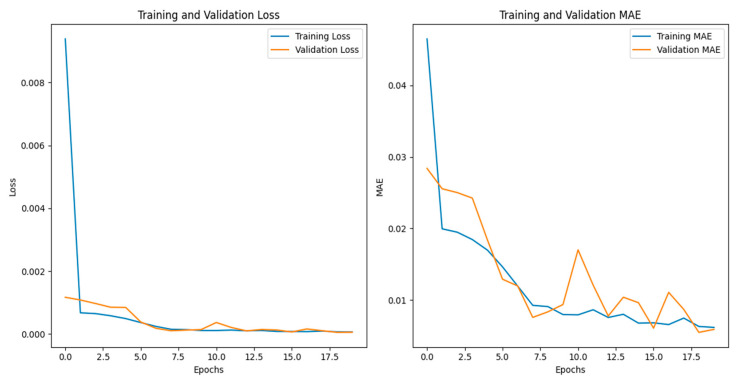
Loss and evaluation of training and validation sets.

In order to assess the efficacy of the model, the mean absolute error (MAE), mean square error (MSE), and the coefficient of determination (R^2^) were calculated as model evaluation indicators. From Figure 14, it can be seen that the mean absolute error of the model was 0.05 °C, the mean square error was 0.05 °C, and the coefficient of determination, R^2^, was 0.9984. Both the mean error and the mean square error were less than 0.1 °C, and the coefficient of determination was high, indicating that the model predicted more accurate results and exhibited a high degree of fit. This suggested that the model was capable of effectively predicting the temperature change of the soil in the greenhouse.

**Figure 15:**
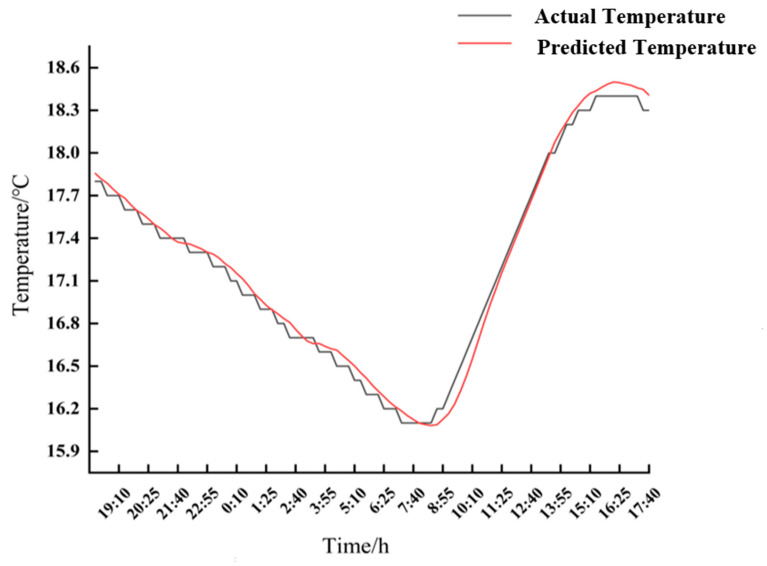
Actual and predicted values for the time period 21 January 2024 18:00–22 January 2024 18:00.

A temperature prediction was conducted at the measuring point, situated at a distance of 2600 mm and a depth of 150 mm within the solar greenhouse. The experimental values for the time period from 18:00 on 21 January 2024 to 18:00 on 22 January 2024, the date when the outdoor temperature was below 20 °C in 2024, were selected and compared with the predicted values of the LSTM prediction model, which included two time periods of covering the greenhouse film and receiving the solar radiation. The principal hyperparameters of the LSTM model were the model structure, optimiser parameters, and the number of training rounds. The number of LSTM units in the first layer of the model structure was 50, and the number of LSTM units in the second layer was 50. The optimiser utilised Adam with a learning rate of 0.001. The number of training rounds (epochs) was 20, the batch size was 32, and the ratio of the dataset, test set, and validation set was 8:1:1.The results are shown in Figure 15.

A comparison of the experimental and model prediction results indicates that the soil temperature decreased over time during the period of covering the trellis film. The maximum temperature difference between the experimental and predicted temperatures was 0.12 °C, with a minimum error of 0.04 °C. During the period of solar radiation receipt, the temperature of the air exhibited a significant increase, reaching a peak, after which there were subsequent fluctuations. The difference between the test temperature and the predicted one was 0.17 °C, with the minimum difference between the two being 0.14 °C. Additionally, the discrepancy between the two temperatures was relatively minor. Therefore, the model prediction results were deemed to be more accurate.

As illustrated by the preceding results, the prediction outcomes were generally satisfactory. Consequently, the LSTM model can be employed to provide an early warning for low temperature control. The model was capable of forecasting the value and time of a low-temperature event, thereby providing the means for precise control of greenhouse soil temperatures and preventing frost damage to crops.

## 4. Conclusions

In this paper, the soil thermal environment of solar greenhouses in Jinzhong, Shanxi Province, China, was zoned. The soil zoning boundary points and the minimum value of temperature in each zone in different months of the overwintering period were defined. The results were as follows:

(1) A mathematical model of solar radiation was developed to theoretically explain the mechanism of soil temperature change.
(2) According to the trend of soil temperature changes, the soil temperature changes were adjusted and divided into three temperature zones: a low-temperature variable temperature zone, a constant-temperature zone, and a high-temperature variable temperature zone. The location of the marginal effect boundary on the south side varied with the season, and the minimum value of the distance between the termination point of the low-temperature region and the south footing was 2480, corresponding to a minimum soil temperature of 9.33 °C.
(3) Throughout the overwintering period, the soil in the low-temperature area of the common solar greenhouse in Jinzhong, Shanxi, China should be heated, and the horizontal distance between this point and the bottom foot of the south side was about 2.6 m. The presence of the film makes the soil temperature inside and outside the greenhouse obviously different, and the better insulation effect of the soil made the soil temperature inside the greenhouse closer to the north wall temperature, which was higher.
(4) In the greenhouse, inside and outside the four positions of different depths of the soil temperature change law, which has been analysed, the soil can be divided into three changes in layer, with the first layer being 0–50 mm deep, where the influence of air temperature on the surface of the soil was greater. The second layer was 50–350 mm, and this layer of soil was partly influenced by air convection, but also, through the deeper layers of soil, by heat transfer, meaning that the temperature change was more moderate. The third layer was 350–450 mm deep, and this layer of soil did not show large fluctuations due to the influence of light intensity and maintained a relatively stable temperature conductivity.
(5) The reliability of the established LSTM model provided a reference basis for greenhouse soil regulation under extreme weather conditions.

## Figures and Tables

**Table 1:** Initial temperature values for each soil boundary.

Location	−10,000 m < y < 0 mm	0 cm < y < 2100 mm	2100 mm < y < 7200 mm	7200 mm < y < 10,200 mm
x = 50 mm	5 °C	14.1 °C	16.4 °C	16.6 °C
x = 250 mm	9 °C	16 °C	18.2 °C	18.3 °C
x = 450 mm	10 °C	16.3 °C	18.4 °C	19.3 °C

**Table 2:** Thermophysical properties of materials.

Parametric	Densities	Specific Heat Capacity	Thermal Conductivity	Absorption Coefficient	Scattering Coefficient	Refractive Index
quilt	70	1880	0.04	0.1	0	1.72
Soil wall	2000	1050	0.8	0.88	0.12	1.92
Soil	1600	1050	0.75	0.88	0.12	1.92
backslope	600	2500	0.29	0.7	0	1.72

**Table 3:** The soil’s low- and high-temperature thresholds in different months.

	December 2023	January 2024	February 2024	March 2024
Low-temperature zone cut-off point	2600	2600	2600	2600
High-temperature zone starting point	9400	8400	8000	8600
Minimal temperature in low-temperature zone	11.23	11.06	11.32	12.65
Maximal temperature in low-temperature zone	18.81	19.05	18.72	18.09
Minimal temperature in constant-temperature zone	19	19.16	19.35	18.29
Minimal temperature in high-temperature zone	19.82	19.66	19.36	19.29

## Data Availability

Data are available upon request from the authors.
